# Development of interpretable machine learning models for predicting the probability of sepsis in patients with pulmonary fibrosis in the intensive care unit: based on MIMIC-IV and multi-database validation

**DOI:** 10.3389/fcimb.2026.1894489

**Published:** 2026-09-07

**Authors:** Yuwei Xia, Junchao Yang

**Affiliations:** The First Affiliated Hospital of Zhejiang Chinese Medical University (Zhejiang Provincial Hospital of Chinese Medicine), Hangzhou, Zhejiang, China

**Keywords:** machine learning, multi-database validation, pulmonary fibrosis, sepsis, SHAP analysis, web deployment

## Abstract

**Background:**

Limited by small sample size, single-institution design, and insufficient comprehensive external validation across heterogeneous healthcare systems, no study to date has systematically validated the predictive performance of machine learning models for sepsis occurrence in an intensive care unit (ICU) population with concomitant pulmonary fibrosis through multiple large-scale databases.

**Methods:**

This retrospective multi-database study utilized two large databases to establish and validate a machine learning model for predicting the probability of sepsis occurrence in ICU patients with pulmonary fibrosis. In this study, 542 patients from the MIMIC-IV database were divided into a training set (381 patients) and an internal validation set (161 patients) in a 7:3 ratio, and external validation was performed on the MIMIC-III (186 patients) database. Six machine learning algorithms were employed: Decision Tree (DT), Extreme Gradient Boosting (XGBoost), Logistic Regression (LR), Lightweight Gradient Boosting Machine (LightGBM), Support Vector Machine (SVM), and Artificial Neural Network (ANN). Baseline variables were screened using least absolute shrinkage and selection operator (Lasso) regression to identify potential predictors. The interpretability of the model was evaluated using Shapley Additive Explanations (SHAP) analysis.

**Results:**

The entire cohort consisted of 728 ICU patients with pulmonary fibrosis. We identified nine consistently crucial clinical characteristics, including gender, dementia, pneumonia, antibiotics, nephrotoxic drugs, glucocorticoids, sequential organ failure assessment (Sofa) score, red blood cell distribution width, and total serum calcium. The ANN algorithm performed optimally, with an area under the curve (AUC) of 0.878 in the training set, 0.837 in the internal validation set, and 0.857 in the MIMIC-III external validation set. SHAP analysis indicated that Sofa was the most influential predictor, followed by antibiotics and pneumonia. Additionally, a web tool was developed to facilitate the prediction of sepsis probability in clinical practice.

**Conclusions:**

This study is the first to develop and validate a machine learning model for predicting sepsis in ICU patients with pulmonary fibrosis across multiple databases. The ANN model, combined with SHAP interpretability, provides a reliable decision-making tool for clinical decision support, and its consistency has been verified in two databases, including our internal validation cohort.

## Introduction

Pulmonary fibrosis (PF) is a group of progressive fibrosing interstitial lung diseases, clinically characterized by progressive dyspnea and irreversible loss of lung function (Bassi et al., 2024). Idiopathic pulmonary fibrosis (IPF) is the subtype with the worst prognosis, with a global incidence estimated at 5.8 per 100, 000 population (Golchin et al., 2025) and a median survival of only 2–4 years after diagnosis (Manolescu et al., 2018), representing the poorest outcome among all non-malignant chronic lung diseases (Karampitsakos et al., 2023). Notably, a considerable proportion of PF patients experience acute exacerbations during the disease course, manifested as rapid deterioration of respiratory function and diffuse alveolar damage, with an annual incidence of up to 9% (Wang et al., 2024), often requiring transfer to the intensive care unit (ICU) for advanced life support (Ali and Glassberg, 2025). Sepsis, a life-threatening organ dysfunction caused by a dysregulated host response to infection (Ansari Khoushabar and Ghafariasl, 2024), is one of the most common fatal complications in ICU patients (La Via et al., 2025). Globally, there are approximately 48.9 million sepsis cases annually, with about 11 million deaths, accounting for 19.7% of all global deaths (Rudd et al., 2020). For critically ill patients with pre-existing pulmonary fibrosis, the superimposition of sepsis undoubtedly exacerbates the situation—the pulmonary structural remodeling and immune system impairment in PF patients severely limit the body’s compensatory capacity against infectious insults (Segal and Molyneaux, 2019), making it highly prone to progression from local infection to systemic inflammatory response and multi-organ dysfunction (Emura and Usuda, 2016). A large-scale study based on the National Inpatient Sample (NIS) demonstrated that IPF patients with sepsis had significantly increased requirements for invasive mechanical ventilation and mortality (Yusuf et al., 2022). This suggests that sepsis is not only highly prevalent in critically ill patients with concomitant pulmonary fibrosis but also significantly worsens prognosis. Therefore, the development of an early prediction tool for sepsis risk in this high-risk population has clear clinical urgency and practical value.

In the field of critical care prediction, machine learning technology has emerged as a powerful medical prediction tool by virtue of its ability to automatically extract features from high-dimensional datasets and capture complex nonlinear associations among variables (Lopes et al., 2025). Compared with traditional statistical models, machine learning-based methods have demonstrated superior performance in predicting the prognosis of various pulmonary and infectious diseases (Lv et al., 2024; Leonard et al., 2025). However, no predictive model for sepsis risk in IPF patients has yet been developed and validated.

To fill the above research gap, this study constructed and validated multiple machine learning models using two large critical care databases to predict the probability of sepsis occurrence in ICU patients with pulmonary fibrosis. In terms of model interpretability, Shapley Additive Explanations (SHAP) analysis was used to provide a visual interpretation of the prediction logic of the optimal model, offering clinicians an intuitive and trustworthy basis for decision-making. This study aims to provide a reliable tool for clinical decision support to explore a new model of early clinical intervention for critically ill patients with pulmonary fibrosis.

## Methods

### Data source

The clinical data sources of all pulmonary fibrosis patients in this study were: the MIMIC-IV (https://mimic.mit.edu/) v3.1 database (Johnson et al., 2023) and the MIMIC-III (https://mimic.mit.edu/) database (Goldberger et al., 2000). The MIMIC-IV database is a comprehensive dataset developed and maintained by the Laboratory for Computational Physiology at the Massachusetts Institute of Technology, which encompasses medical information related to patients admitted to the intensive care units of the Beth Israel Deaconess Medical Center. The MIMIC-III CareVue subset database was used as a same-institution historical external validation cohort, which avoided potential patient-level overlap between the MIMIC-IV development cohort and the MIMIC-III validation cohort. One author completed the Collaborative Institutional Training Initiative (CITI) (Record ID 73874649) and obtained access to the MIMIC-IV and MIMIC-III databases.

### Participants

(1) patients diagnosed with pulmonary fibrosis based on ICD codes; (2) age between 18 and 100 years; (3) ICU length of stay ≥ 24 hours; (4) first ICU admission. Patients with more than 20% missing data were excluded from the analysis. The diagnosis of sepsis was based on the Sepsis-3 diagnostic criteria, and enrolled patients were divided into sepsis group and non-sepsis group according to the occurrence of sepsis.

### Variable extraction and data preprocessing

A total of 90 candidate predictor variables were initially extracted from the MIMIC-IV and MIMIC-III databases. Candidate predictors were selected based on clinical relevance, routine availability within the early ICU period, and prior evidence related to infection, organ dysfunction, comorbidity burden, medication exposure, and laboratory abnormalities in critically ill patients with pulmonary fibrosis. Variables with more than 20% missing values were excluded before multiple imputation. During this initial screening step, 33 candidate predictor variables were excluded, and the remaining 57 candidate predictors were retained for subsequent multiple imputation and model development.

The retained candidate predictors included age, gender, weight, language, insurance, marital status, heart rate, temperature, respiratory rate, systolic blood pressure (SBP), diastolic blood pressure (DBP), mean blood pressure (MBP), hematocrit, red blood cell distribution width (RDW), red blood cell count (RBC), hemoglobin, white blood cell count (WBC), platelet count, anion gap, calcium, potassium, sodium, chloride, glucose, creatinine, international normalized ratio (INR), prothrombin time (PT), partial thromboplastin time (PTT), hypertension, chronic kidney disease, acute renal failure, liver cirrhosis, hepatitis, pneumonia, tuberculosis, chronic bronchitis, cerebrovascular accident, diabetes mellitus, malignancy, hyperlipidemia, ischemic heart disease, myocardial infarction, heart failure, peptic ulcer disease, dementia, asthma, chronic obstructive pulmonary disease (COPD), mechanical ventilation, glucocorticoids, immunosuppressants, vasopressors, antibiotics, nephrotoxic drugs, neuromuscular blocking agents, Sequential Organ Failure Assessment (SOFA) score, Simplified Acute Physiology Score II (SAPS II), and Charlson Comorbidity Index (Charlson). For vital signs and laboratory tests, the first recorded values within the first 24 hours after ICU admission were used.

After variable-level missingness screening, patients with more than 20% missing data among the retained candidate predictors were further excluded from the final cohort. Missing data were handled using multiple imputation by chained equations (MICE) package in R. Variables with more than 20% missing values were excluded before imputation. For the remaining candidate predictors, five imputed datasets were generated with a random seed of 3407 to ensure reproducibility.

The actual imputation methods were checked using “data_imputed$method”. Logistic regression (logreg) was used for binary variables with missing values, including Medicare and English; polytomous logistic regression (polyreg) was used for the unordered categorical variable Marital_status; and predictive mean matching (pmm) was used for continuous variables with missing values, including Weight, Temperature, SBP, DBP, MBP, Hematocrit, Platelet_count, RDW, RBC, WBC, Calcium_total, INR, PT, and PTT. Variables without missing values were not imputed.

The outcome variable was not imputed. Patients with missing outcome information were excluded during the study population selection process before multiple imputation; therefore, all included patients had complete outcome data.

### Feature selection

MIMIC-IV was randomly divided into a training set (70%) and an internal validation set (30%). Stratified sampling was performed using the createDataPartition function in R to ensure balanced class distributions in the training and validation sets for subsequent model evaluation. No additional class-balancing techniques, such as SMOTE, class weighting, or undersampling, were applied during model training to preserve the original cohort distribution and avoid introducing an artificially balanced training distribution through resampling. The least absolute shrinkage and selection operator (Lasso) algorithm was used to screen baseline variables to identify potential predictors in the training set. Lasso is a linear regression-based model that regularizes regression coefficients by imposing an L1 penalty. To find the optimal constraint parameter, Lasso regression combined with 10-fold cross-validation was used for variable selection.

### Model establishment and comparison

Six machine learning algorithms were implemented using Python 3.10: Decision Tree (DT) (Lemon et al., 2003), Extreme Gradient Boosting (XGBoost) (Chen and Guestrin, 2016), Logistic Regression (LR) (Shipe et al., 2019), Light Gradient Boosting Machine (LightGBM) (Ke et al., 2017), Support Vector Machine (SVM) (Cortes and Vapnik, 1995), and Artificial Neural Network (ANN) (Shahid et al., 2019). To ensure model stability, 10-fold cross-validation and grid search were used for hyperparameter optimization. Model performance was compared using multiple metrics including AUC, accuracy, sensitivity, specificity, F1 score, and confusion matrices. The receiver operating characteristic (ROC) curves of the training set and validation set were compared, and calibration curves and decision curve analysis (DCA) were performed on the validation set to evaluate the clinical utility of each model.

### Sensitivity analysis

Given that SOFA score, pneumonia, and antibiotics are related to the clinical management of sepsis, additional sensitivity analyses were performed to evaluate the potential influence of these variables on model performance. Specifically, we constructed sensitivity models by excluding SOFA score, pneumonia, antibiotics, and all three variables simultaneously from the candidate predictor set. After each exclusion strategy, the complete model development procedure, including LASSO-based feature selection, model training, hyperparameter tuning, and validation, was repeated. The performance of the sensitivity models was evaluated using the AUC, and the results were compared with those of the primary model.

### Model interpretation

The Shapley Additive Explanations (SHAP) method was used to investigate the contribution of each feature variable to the final output of the model. SHAP analysis utilizes game theory approaches, compensates for the black-box characteristics of high-performance machine learning models, focuses on blind spots in complex information, and significantly enhances model interpretability (Linardatos et al., 2020).

### Statistical analysis

R software (version 4.5.1) was used for statistical analysis. Baseline statistics for all variables were first calculated, and the Shapiro-Wilk test was used to assess the normality of continuous variables. Normally distributed continuous data were analyzed using t-tests; non-normally distributed continuous data were analyzed using the Wilcoxon rank-sum test. Categorical variables were compared using the chi-square test. A p-value < 0.05 was considered statistically significant.

## Results

### Baseline characteristics

**Figure 1:**
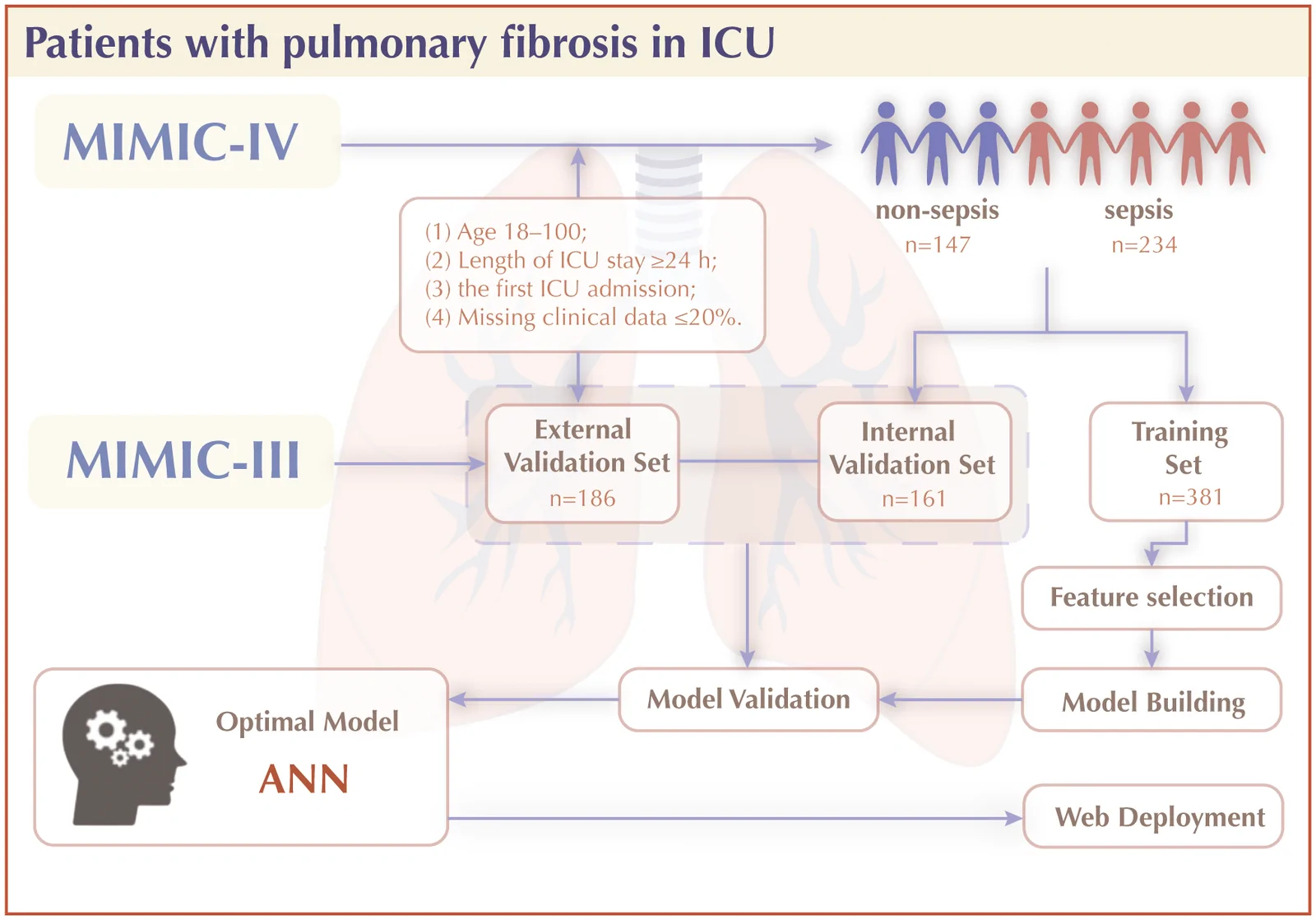
Workflow diagram. MIMIC-IV, Medical Information Mart for Intensive Care IV; MIMIC-III, Medical Information Mart for Intensive Care III.

The patient selection process from the databases is shown in **Figure 1**. After excluding patients with non-first ICU admission, age <18 or >100 years, ICU length of stay <24 hours, and those with >20% missing individual data, a total of 728 patients with pulmonary fibrosis were finally included: 381 from MIMIC-IV (training dataset), 161 from MIMIC-IV (internal validation dataset), and 186 from MIMIC-III (external validation dataset). The outcome of this model focused on the probability of sepsis occurrence in ICU patients with pulmonary fibrosis. In the MIMIC-IV training set, 147 patients had negative outcomes (non-sepsis group) and 234 patients had positive outcomes (sepsis group). Baseline analyses were performed with a focus on the outcome of sepsis occurrence (**Table 1**).

**Table 1:** Baseline table.

Variable	Overall (n=381)	Non-sepsis (n=147)	Sepsis (n=234)	*p*
Gender n (%)				0.622
Female	155 (40.68)	57 (38.78)	98 (41.88)	
Male	226 (59.32)	90 (61.22)	136 (58.12)	
Medicare n (%)				0.698
No	111 (29.13)	45 (30.61)	66 (28.21)	
Yes	270 (70.87)	102 (69.39)	168 (71.79)	
English n (%)				0.468
No	46 (12.07)	15 (10.20)	31 (13.25)	
Yes	335 (87.93)	132 (89.80)	203 (86.75)	
Marital status n (%)				0.848
Married	96 (25.20)	37 (25.17)	59 (25.21)	
Single	205 (53.81)	77 (52.38)	128 (54.70)	
Others	80 (21.00)	33 (22.45)	47 (20.09)	
Peptic ulcer disease n (%)				1.000
No	366 (96.06)	141 (95.92)	225 (96.15)	
Yes	15 (3.94)	6 (4.08)	9 (3.85)	
Dementia n (%)				0.362
No	356 (93.44)	140 (95.24)	216 (92.31)	
Yes	25 (6.56)	7 (4.76)	18 (7.69)	
Asthma n (%)				0.390
No	345 (90.55)	136 (92.52)	209 (89.32)	
Yes	36 (9.45)	11 (7.48)	25 (10.68)	
Hypertension n (%)				1.000
No	222 (58.27)	86 (58.50)	136 (58.12)	
Yes	159 (41.73)	61 (41.50)	98 (41.88)	
Acute kidney injury n (%)				0.013
No	228 (59.84)	100 (68.03)	128 (54.70)	
Yes	153 (40.16)	47 (31.97)	106 (45.30)	
Cirrhosis n (%)				1.000
No	368 (96.59)	142 (96.60)	226 (96.58)	
Yes	13 (3.41)	5 (3.40)	8 (3.42)	
Hepatitis n (%)				1.000
No	374 (98.16)	144 (97.96)	230 (98.29)	
Yes	7 (1.84)	3 (2.04)	4 (1.71)	
Pulmonary tuberculosis n (%)				1.000
No	368 (96.59)	142 (96.60)	226 (96.58)	
Yes	13 (3.41)	5 (3.40)	8 (3.42)	
Pneumonia n (%)				<0.001
No	216 (56.69)	105 (71.43)	111 (47.44)	
Yes	165 (43.31)	42 (28.57)	123 (52.56)	
Cerebrovascular accident n (%)				0.715
No	346 (90.81)	135 (91.84)	211 (90.17)	
Yes	35 (9.19)	12 (8.16)	23 (9.83)	
Chronic kidney disease n (%)				0.735
No	288 (75.59)	113 (76.87)	175 (74.79)	
Yes	93 (24.41)	34 (23.13)	59 (25.21)	
Malignant tumor n (%)				0.192
No	319 (83.73)	118 (80.27)	201 (85.90)	
Yes	62 (16.27)	29 (19.73)	33 (14.10)	
Diabetes n (%)				0.743
No	275 (72.18)	108 (73.47)	167 (71.37)	
Yes	106 (27.82)	39 (26.53)	67 (28.63)	
Hyperlipidemia n (%)				0.655
No	237 (62.20)	94 (63.95)	143 (61.11)	
Yes	144 (37.80)	53 (36.05)	91 (38.89)	
Chronic bronchitis n (%)				1.000
No	331 (86.88)	128 (87.07)	203 (86.75)	
Yes	50 (13.12)	19 (12.93)	31 (13.25)	
Heart failure n (%)				0.210
No	201 (52.76)	84 (57.14)	117 (50.00)	
Yes	180 (47.24)	63 (42.86)	117 (50.00)	
Myocardial infarction n (%)				0.486
No	366 (96.06)	143 (97.28)	223 (95.30)	
Yes	15 (3.94)	4 (2.72)	11 (4.70)	
Ischemic heart disease n (%)				0.987
No	224 (58.79)	87 (59.18)	137 (58.55)	
Yes	157 (41.21)	60 (40.82)	97 (41.45)	
COPD n (%)				0.481
No	247 (64.83)	99 (67.35)	148 (63.25)	
Yes	134 (35.17)	48 (32.65)	86 (36.75)	
Antibiotics n (%)				<0.001
No	52 (13.65)	51 (34.69)	1 (0.43)	
Yes	329 (86.35)	96 (65.31)	233 (99.57)	
Neuromuscular blockers n (%)				0.054
No	365 (95.80)	145 (98.64)	220 (94.02)	
Yes	16 (4.20)	2 (1.36)	14 (5.98)	
Vasopressin n (%)				<0.001
No	212 (55.64)	107 (72.79)	105 (44.87)	
Yes	169 (44.36)	40 (27.21)	129 (55.13)	
Immunosuppressant n (%)				0.393
No	354 (92.91)	134 (91.16)	220 (94.02)	
Yes	27 (7.09)	13 (8.84)	14 (5.98)	
Neurotoxic drugs n (%)				0.002
No	119 (31.23)	60 (40.82)	59 (25.21)	
Yes	262 (68.77)	87 (59.18)	175 (74.79)	
Glucocorticoid n (%)				0.045
No	210 (55.12)	91 (61.90)	119 (50.85)	
Yes	171 (44.88)	56 (38.10)	115 (49.15)	
Ventilation n (%)				0.133
No	37 (9.71)	19 (12.93)	18 (7.69)	
Yes	344 (90.29)	128 (87.07)	216 (92.31)	
Age [years, median (IQR)]	75.00 [66.00, 82.00]	75.00 [66.00, 82.00]	74.00 [65.25, 82.00]	0.716
Weight [kg, median (IQR)]	74.90 [63.50, 87.60]	76.00 [64.75, 89.50]	74.05 [63.00, 87.25]	0.488
Heartrate [times/minute, median (IQR)]	89.00 [78.00, 105.00]	89.00 [74.50, 101.50]	89.00 [79.00, 107.00]	0.105
Respiratory rate [times/minute, median (IQR)]	21.00 [16.00, 27.00]	20.00 [17.00, 26.00]	21.50 [16.00, 27.00]	0.374
Temperature [℉, median (IQR)]	98.00 [97.60, 98.60]	98.00 [97.50, 98.50]	98.10 [97.60, 98.70]	0.127
SBP [mmHg, median (IQR)]	121.00 [105.00, 138.00]	126.00 [111.00, 140.00]	118.50 [102.00, 137.00]	0.022
DBP [mmHg, median (IQR)]	65.00 [56.00, 78.00]	67.00 [58.00, 80.00]	63.00 [54.00, 76.00]	0.006
MBP [mmHg, median (IQR)]	79.00 [69.00, 92.00]	80.00 [72.50, 93.00]	76.50 [67.00, 89.00]	0.003
Sofa [median (IQR)]	4.00 [2.00, 6.00]	2.00 [1.00, 4.00]	5.00 [3.00, 7.00]	<0.001
Sapsii [median (IQR)]	35.00 [29.00, 44.00]	32.00 [27.00, 40.00]	37.00 [31.00, 46.00]	<0.001
Charlson [median (IQR)]	6.00 [4.00, 8.00]	5.00 [4.00, 7.00]	6.00 [5.00, 8.00]	0.071
Hematocrit [%, median (IQR)]	31.80 [28.00, 36.60]	32.60 [28.50, 37.25]	31.30 [27.25, 35.48]	0.031
Hemoglobin [g/dL, median (IQR)]	10.40 [9.00, 11.80]	10.60 [9.40, 12.25]	10.00 [8.70, 11.60]	0.007
Platelet count [109/L, median (IQR)]	209.00 [141.00, 287.00]	223.00 [163.50, 278.50]	198.00 [130.25, 290.00]	0.100
RDW [%, median (IQR)]	15.00 [13.90, 16.50]	14.80 [13.60, 16.10]	15.25 [14.00, 16.70]	0.086
RBC [10^12^/L, median (IQR)]	3.49 [3.04, 3.94]	3.59 [3.06, 4.06]	3.47 [2.98, 3.88]	0.041
WBC [10^9^/L, median (IQR)]	11.10 [8.30, 15.20]	10.10 [8.10, 14.45]	11.70 [8.62, 15.57]	0.088
Anion gap [mEq/L, median (IQR)]	14.00 [11.00, 16.00]	13.00 [11.00, 15.00]	14.00 [12.00, 17.00]	0.046
Calcium total [mg/dL, median (IQR)]	8.40 [8.00, 8.90]	8.60 [8.20, 8.95]	8.30 [7.80, 8.80]	<0.001
Chloride [mEq/L, median (IQR)]	103.00 [98.00, 107.00]	103.00 [99.00, 106.00]	103.00 [98.00, 107.00]	0.273
Glucose [mg/dL, median (IQR)]	129.00 [106.00, 166.00]	127.00 [105.50, 157.00]	131.00 [107.00, 170.50]	0.454
Potassium [mEq/L, median (IQR)]	4.10 [3.80, 4.50]	4.20 [3.80, 4.60]	4.10 [3.80, 4.50]	0.772
Sodium [mEq/L, median (IQR)]	138.00 [135.00, 141.00]	138.00 [136.00, 141.00]	138.00 [135.00, 140.75]	0.163
Creatinine [mg/dL, median (IQR)]	1.00 [0.70, 1.40]	1.00 [0.70, 1.30]	1.10 [0.72, 1.60]	0.033
INR [median (IQR)]	1.30 [1.20, 1.60]	1.30 [1.10, 1.50]	1.30 [1.20, 1.60]	0.051
PT [seconds, median (IQR)]	14.20 [12.90, 17.40]	14.00 [12.50, 16.50]	14.40 [13.00, 17.88]	0.056
PTT [seconds, median (IQR)]	30.80 [27.60, 36.30]	30.60 [27.95, 35.85]	31.10 [27.50, 36.68]	0.824

### Feature selection

**Figure 2:**
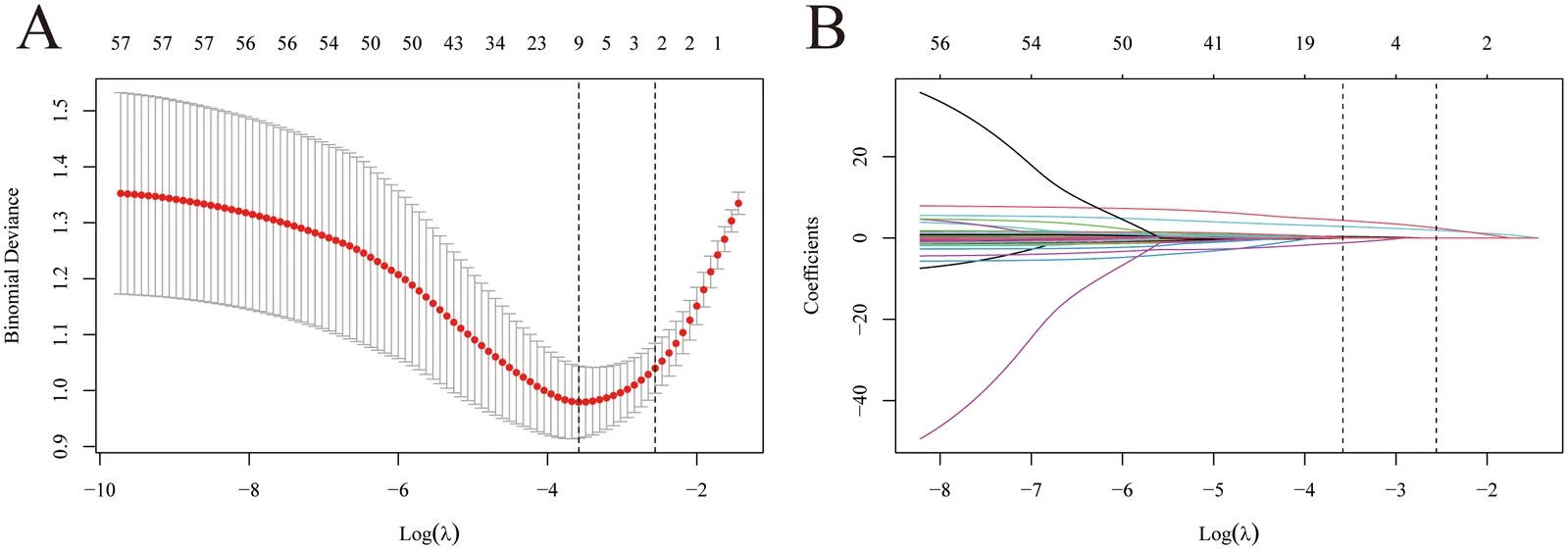
Feature selection results of Lasso regression. **(A)** Optimal λ selection in Lasso regression using 10-fold cross-validation. The misclassification error for different variables with respect to log(λ) is shown. The two vertical dashed lines indicate the optimal values under the minimum and 1-standard error (1-SE) criteria, respectively. “λ” is the tuning parameter, and a total of nine predictors with non-zero coefficients were identified. **(B)** Distribution of Lasso regression coefficients for all baseline features.

In the training set, the Lasso algorithm with 10-fold cross-validation yielded a minimum λ value of 0.028, and nine variables were screened as predictors for establishing the machine learning prediction models at this value (**Figures 2A, B**). These variables included gender, dementia, pneumonia, antibiotics, nephrotoxic drugs, glucocorticoids, Sequential Organ Failure Assessment (SOFA) score, red blood cell distribution width, and total serum calcium.

### Model development and performance evaluation

Based on the nine important feature variables identified by Lasso regression, six machine learning models were developed and evaluated for their ability to predict the probability of sepsis occurrence in pulmonary fibrosis patients. After defining hyperparameter search ranges and performing grid search with GridSearchCV and 10-fold cross-validation, the optimal hyperparameter combinations were obtained, as detailed in **Table 2**.

**Table 2:** Optimal hyperparameter combinations for machine learning models.

Models	Optimal hyperparameter combinations
DT	‘ccp_alpha’: 0.0, ‘max_depth’: 3, ‘max_features’: ‘sqrt’, ‘min_samples_split’: 5
XGBoost	‘learning_rate’: 0.1, ‘max_depth’: 3, ‘n_estimators’: 100, ‘subsample’: 1.0
LightGBM	‘colsample_bytree’: 0.6, ‘learning_rate’: 0.1, ‘n_estimators’: 100, ‘num_leaves’: 31, ‘subsample’: 0.6
SVM	‘C’: 0.1, ‘degree’: 2, ‘gamma’: ‘scale’, ‘kernel’: ‘linear’
ANN	‘activation’: ‘logistic’, ‘hidden_layer_sizes’: (50, 50)

**Figure 3:**
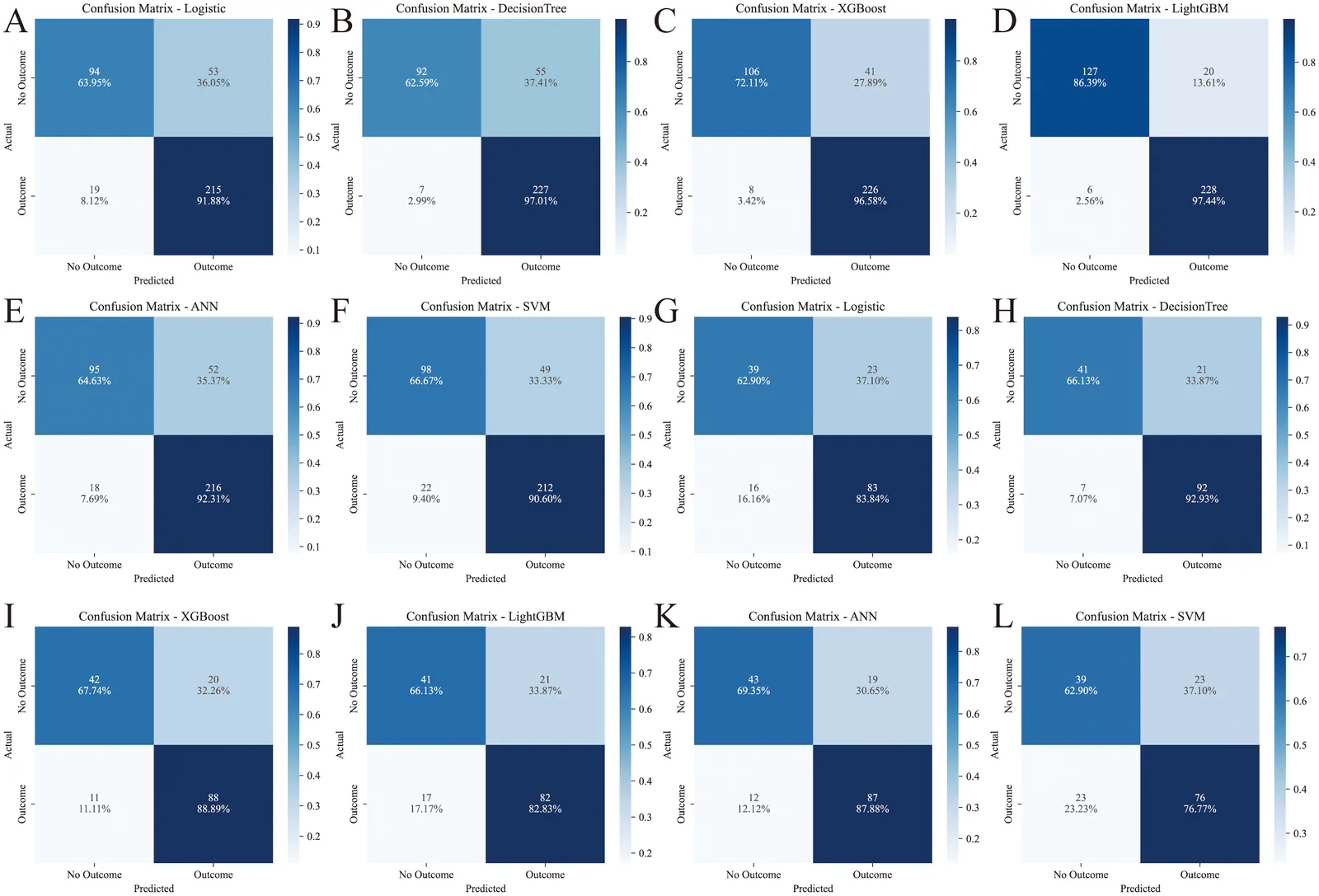
Confusion matrix of training and validation sets. **(A–F)** Confusion matrices of training set. **(A)** Logistic Regression (LR). **(B)** Decision Tree (DT). **(C)** Extreme Gradient Boosting (XGBoost). **(D)** Light Gradient Boosting Machine (LightGBM). **(E)** Artificial Neural Network (ANN). **(F)** Support Vector Machine (SVM). **(G–L)** Confusion matrices of internal validation set. **(G)** LR. **(H)** DT. **(I)** XGBoost. **(K)** ANN. **(L)** SVM.

**Figure 4:**
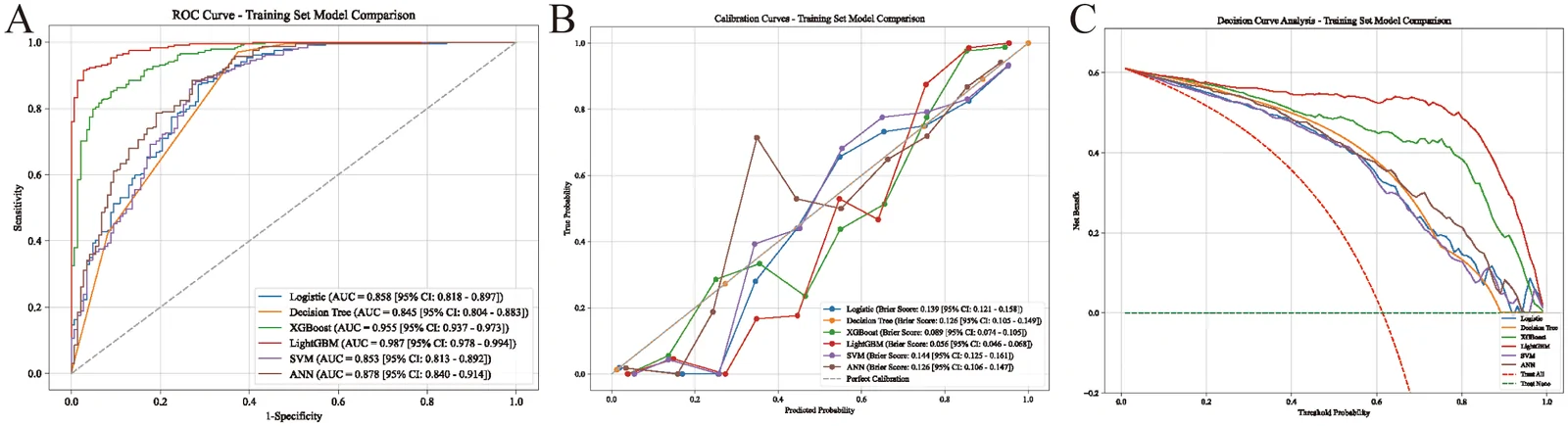
Model evaluation of the training set. **(A)** Receiver Operating Characteristic (ROC). **(B)** Calibration curve. **(C)** Decision Curve Analysis (DCA).

To clarify the performance of each model, various metrics (including AUC, accuracy, sensitivity, specificity, F1 score), confusion matrices (**Figure 3**), receiver operating characteristic (ROC) curves, calibration curves, and decision curve analysis (DCA) were evaluated in the training set and validation set of MIMIC-IV. The performance of each model in the training set is detailed in **Table 3** and **Figure 4**.

**Table 3:** Performance of models in training set.

Models	AUC	AUC 95% CI lower	AUC 95% CI upper	Accuracy	Sensitivity	Specificity	F1 Score
LR	0.858	0.818	0.897	0.811	0.919	0.639	0.857
DT	0.845	0.804	0.883	0.837	0.970	0.626	0.880
XGBoost	0.955	0.937	0.973	0.871	0.966	0.721	0.902
LightGBM	0.987	0.978	0.994	0.932	0.974	0.864	0.946
SVM	0.853	0.813	0.892	0.814	0.906	0.667	0.857
ANN	0.878	0.840	0.914	0.816	0.923	0.646	0.861

**Figure 5:**
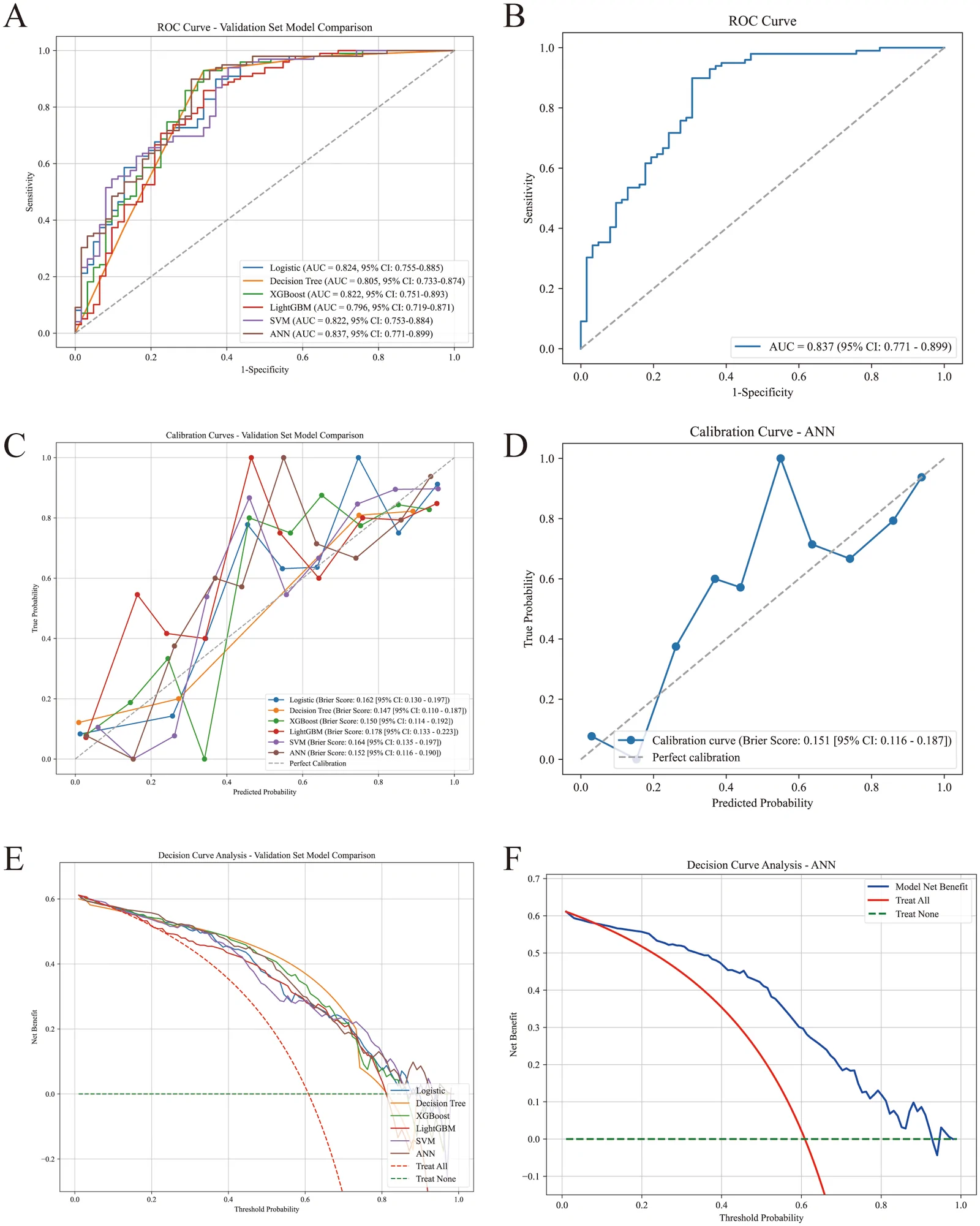
Model evaluation of internal validation set. **(A)** Comparison of ROC curves of six different machine learning models. **(B)** ROC curve of the ANN model. **(C)** Comparison of calibration curves of six different machine learning models. **(D)** Calibration curve of the ANN model. **(E)** Comparison of DCA curves of six different machine learning models. **(F)** DCA curve of the ANN model.

**Figure 6:**
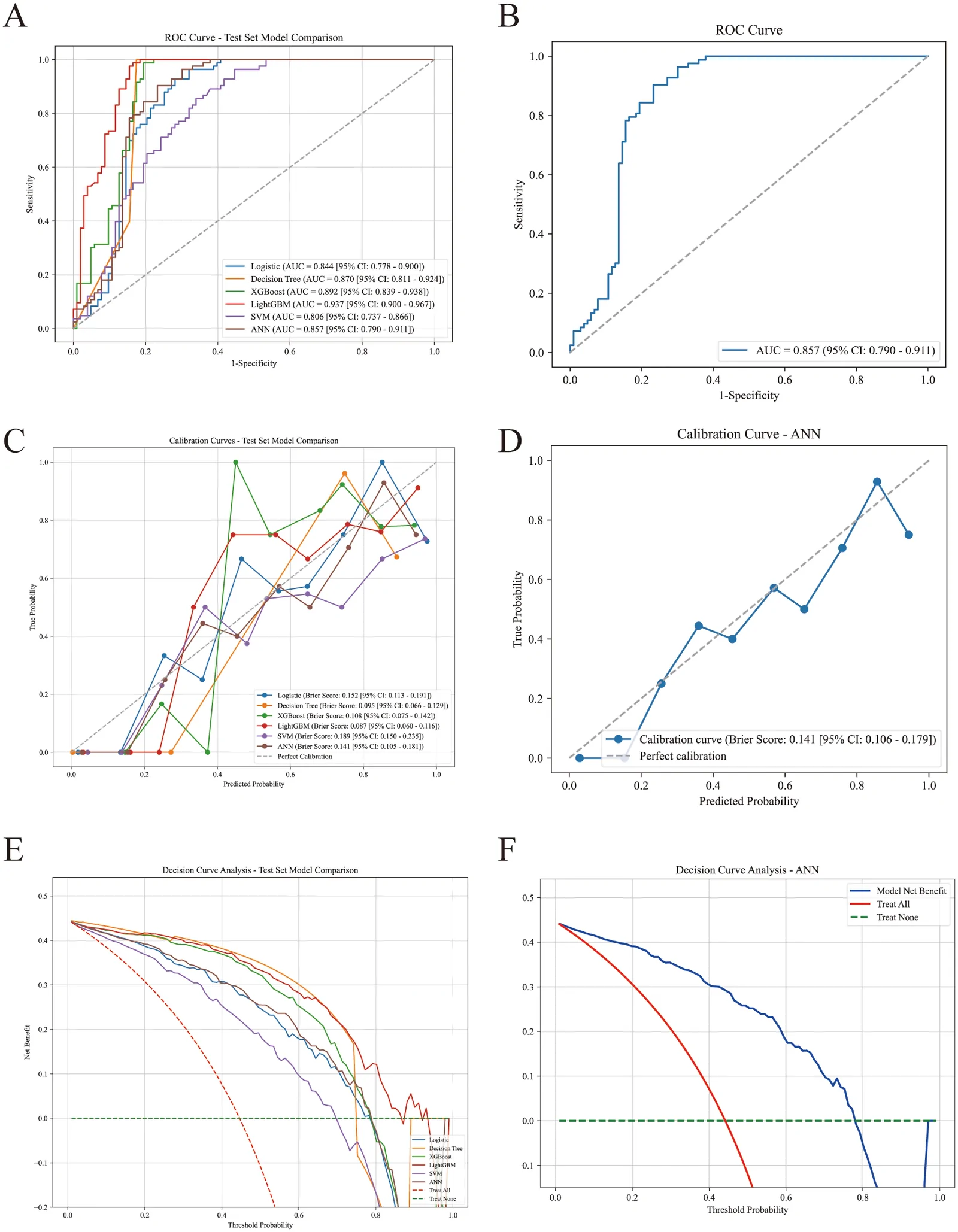
Model evaluation of the external validation set. **(A)** Comparison of ROC curves of six different machine learning models. **(B)** ROC curve of the ANN model. **(C)** Comparison of calibration curves of six different machine learning models. **(D)** Calibration curve of the ANN model. **(E)** Comparison of DCA curves of six different machine learning models. **(F)** DCA curve of the ANN model.

ANN achieved the highest AUC in the internal validation set and showed favorable overall performance across accuracy, sensitivity, specificity and F1 score performance (**Table 4**). Although the AUC differences between ANN and other candidate models were not statistically significant, ANN demonstrated consistently favorable clinical net benefit in both the internal and external validation cohorts. Therefore, ANN was selected as the final model based on integrated performance. In the MIMIC-IV training set, ANN achieved an excellent AUC of 0.878 (95% CI: 0.840 to 0.914) (**Table 3**). In the MIMIC-IV validation set (**Figures 5A, B**) and the MIMIC-III dataset (**Figures 6A, B**), the AUCs of ANN were 0.837 (95% CI: 0.771 to 0.899) and 0.857 (95% CI: 0.790 to 0.911), respectively. Other models also achieved relatively good AUC performance in the validation set: LR (mean AUC: 0.834), DT (mean AUC: 0.837), XGBoost (mean AUC: 0.857), LightGBM (mean AUC: 0.866), and SVM (mean AUC: 0.814).

**Table 4:** Performance of models in internal validation set.

Models	AUC	AUC 95% CI lower	AUC 95% CI upper	Accuracy	Sensitivity	Specificity	F1 Score
LR	0.824	0.755	0.885	0.758	0.838	0.629	0.810
DT	0.805	0.733	0.874	0.826	0.929	0.661	0.868
XGBoost	0.822	0.751	0.893	0.807	0.889	0.677	0.850
LightGBM	0.796	0.719	0.871	0.764	0.828	0.661	0.812
SVM	0.822	0.753	0.884	0.714	0.768	0.629	0.768
ANN	0.837	0.771	0.899	0.807	0.879	0.694	0.849

Calibration curves showed the Brier score for each model. The deviation between model outputs and actual outcomes was well-behaved for all models, with Brier scores ranging from 0.021 to 0.144 in the MIMIC-IV training set (**Figure 4B**) and from 0.147 to 0.178 in the validation set (**Figures 5C, D**). In addition, Brier scores ranged from 0.087 to 0.189 in the external validation set (**Figures 6C, D**).

DCA confirmed the clinical utility of the models. In the internal and external validation sets, the models showed net benefit superior to the treat-all and treat-none strategies, and the model demonstrated an appropriate risk threshold range of 0.60 to 0.95 (**Figures 5E, F**) and 0.45 to 0.90 (**Figures 6E, F**). Among them, ANN achieved the best clinical benefit in both the internal and external validation sets.

The model demonstrated good performance and ROC curves in both internal and external validation sets, exhibiting superior generalizability across multiple databases, thereby providing more instructive decision-making value for clinical assessment of sepsis risk in ICU patients with pulmonary fibrosis.

### Sensitivity analysis

To further assess the robustness of the model and the potential influence of variables related to sepsis clinical management, we conducted a series of sensitivity analyses (**Table 5**). When these variables were excluded individually, the models maintained favorable discrimination in both the training and validation sets. Specifically, after excluding SOFA score, the training and validation AUCs were 0.811 and 0.794, respectively. After excluding pneumonia, the corresponding AUCs were 0.860 and 0.844, respectively. After excluding antibiotics, the training and validation AUCs were 0.820 and 0.798, respectively. The small differences between the training and validation AUCs suggested no substantial overfitting in these sensitivity models.

**Table 5:** Sensitivity analyses excluding variables related to sepsis clinical management.

Sensitivity analysis	Training AUC	Validation AUC	AUC difference
Excluding SOFA score	0.811	0.794	0.017
Excluding pneumonia	0.860	0.844	0.016
Excluding antibiotics	0.820	0.798	0.022
Excluding SOFA score, pneumonia, and antibiotics	0.744	0.703	0.041

In the most conservative analysis, after excluding all three variables simultaneously, the model still retained moderate discriminatory ability, with training and validation AUCs of 0.744 and 0.703, respectively. Although the AUC decreased compared with that of the primary model, the model still retained moderate discriminatory ability. This finding suggests that these variables provided additional predictive information and could further improve model performance, but they were not strictly required for maintaining the model’s predictive ability. Even after complete exclusion of these variables, the model still achieved moderate discrimination. Collectively, these findings provide supportive evidence for the robustness of the overall predictive framework and suggest that the primary model performance was not driven solely by variables related to sepsis clinical management.

### SHAP-based feature importance and dependence analysis

**Figure 7:**
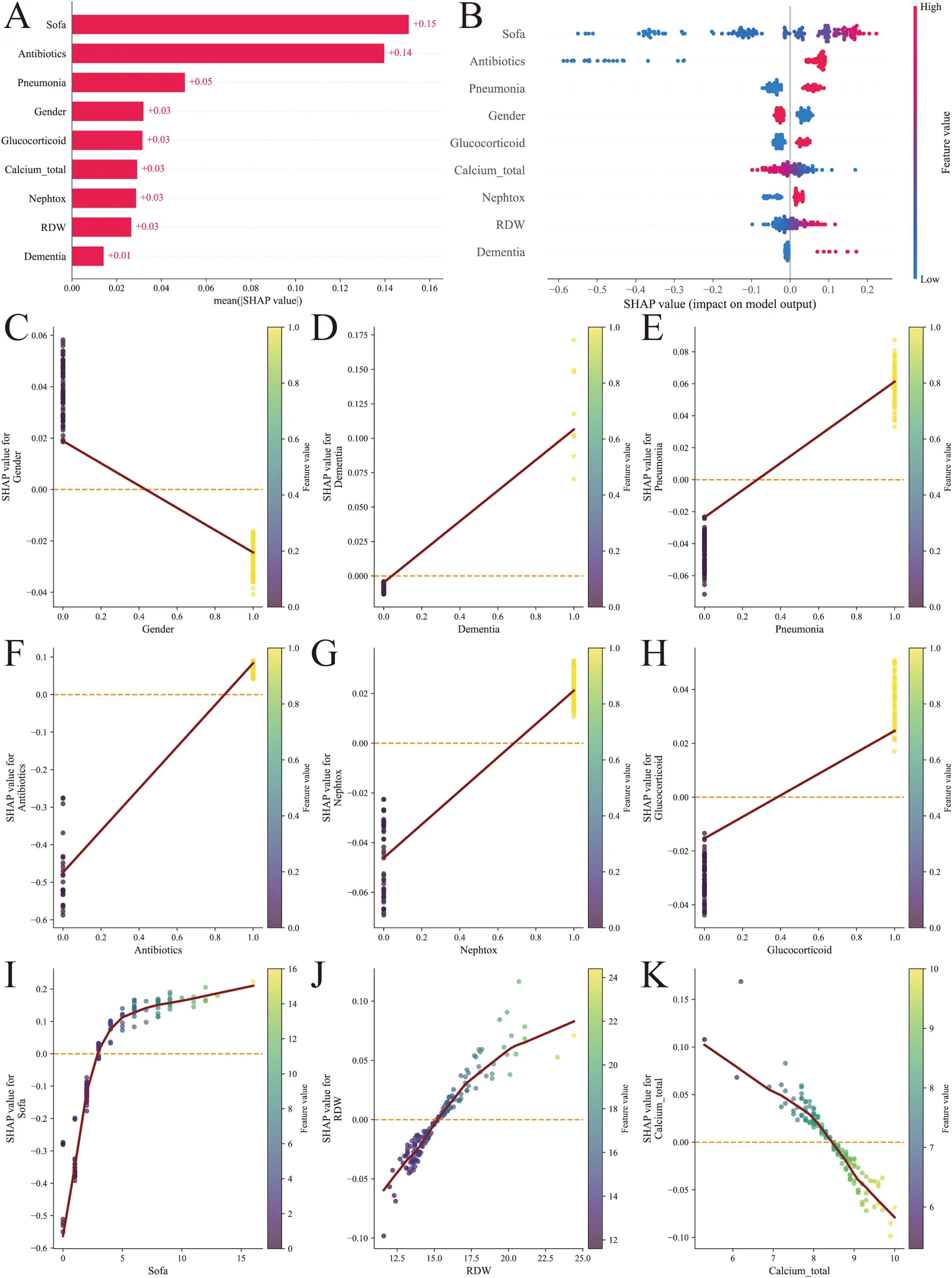
Utilize SHAP analysis to reveal the importance ranking of each key feature variable and its correlation with sepsis risk. **(A)** Ranking of nine important feature variables by SHAP value. **(B)** Bee swarm plot using color gradient to represent positive and negative correlations between feature variables and sepsis risk. **(C–K)** Dependence plots for nine key feature variables, including gender, dementia, pneumonia, antibiotics, nephrotoxic drugs, glucocorticoids, SOFA, RDW, and total serum calcium.

To highlight the interpretability of the relationship between the prediction model and clinical variables, SHAP analysis was introduced to reveal the key factors influencing the probability of sepsis occurrence in the ANN model. **Figure 7A** shows the nine significant feature variables ranked by SHAP values, with the length of the bars representing the magnitude of their respective contributions to the model’s prediction outcomes. Among them, SOFA was the most influential predictor. **Figure 7B** uses a color gradient to represent the positive and negative correlations between feature variables and sepsis risk. Red points are associated with increased risk, while purple points indicate decreased risk. For each feature variable, the more red points on the side with positive SHAP values, the more the larger values tend to increase sepsis risk, and vice versa.

The SHAP dependence plots for each key clinical feature reveal the complex nonlinear relationships between different features and model-predicted outcomes. The horizontal axis represents the feature value, and the vertical axis represents the SHAP value, which quantifies the marginal contribution strength and direction of the feature variable to the prediction outcome. A positive SHAP value (above the yellow line) indicates that the feature has a positive promoting effect on the prediction target, while a negative value indicates an inhibitory effect, with the absolute value directly reflecting the feature’s influence weight. Positive promoting effects were observed when patients were “female” (value=0) and when the feature variables “dementia, “ “pneumonia, “ “antibiotics, “ “nephrotoxic drugs, “ and “glucocorticoids” were “present” (value=1), indicating that the presence of these features promotes an increased risk of sepsis occurrence. Among the continuous variables, the fitted lines for “SOFA” and “RDW” generally showed an upward trend with increasing values on the horizontal axis, indicating that as these variables increase, their contribution to sepsis risk rises. Total serum calcium showed the opposite trend (**Figures 7C–K**). Furthermore, the approximate “promotion” to “inhibition” turning points for these continuous variables were: SOFA (4 points), RDW (15%), and total serum calcium (8.5 mg/dL). The dependence plots, by visualizing the association between feature values and SHAP values, provide intuitive support for understanding the predictive value, direction of effect, and potential mechanisms of each clinical feature, helping to clarify the core driving factors of model decisions.

### Individual patient risk profiling

SHAP waterfall plots and force plots visually expressed the differences in contribution of specific sample sepsis risk features from the training set. In the waterfall plot, each feature variable is represented by a bar, where blue indicates a negative contribution of the variable to the prediction outcome (i.e., reducing sepsis risk), and red indicates the opposite, representing increased sepsis risk. The force plot illustrates how each feature contributes to the final predicted value f(x), starting from the model’s baseline prediction value. Blue arrows indicate decreasing contributions, while red arrows indicate increasing contributions.

**Figure 8:**
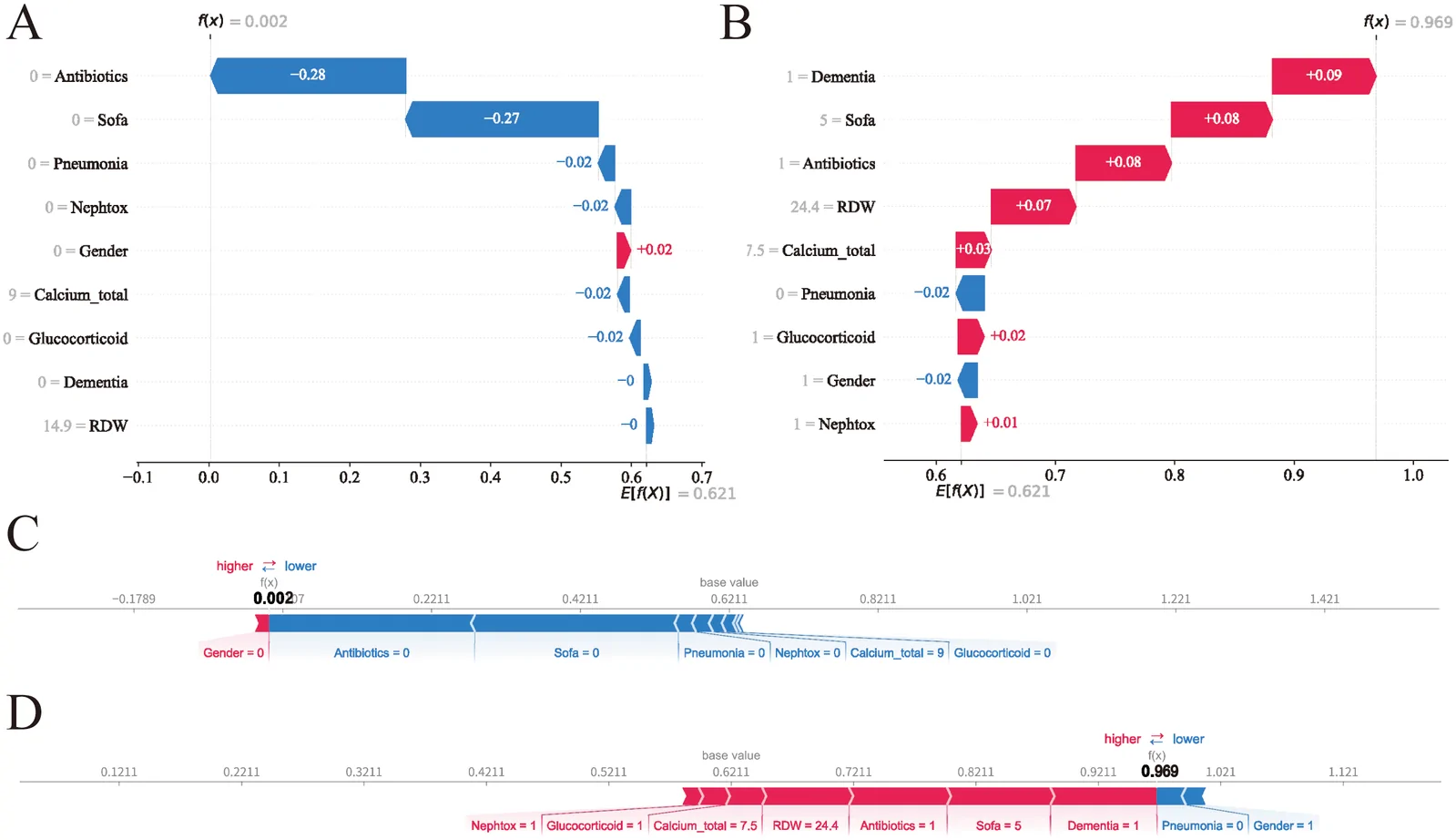
SHAP waterfall plots and force plots for predicting the probability of sepsis in pulmonary fibrosis patients. **(A)** Waterfall plot of a patient without sepsis. **(B)** Waterfall plot of a patient with sepsis. **(C)** Force plot of a patient without sepsis. **(D)** Force plot of a patient with sepsis.

**Figures 8A, C** show the waterfall and force plots for the same patient. Antibiotics (=0), SOFA (=0), pneumonia (=0), nephrotoxic drugs (=0), total serum calcium (=9 mg/dL), glucocorticoids (=0), dementia (=0), and RDW (=14.9%) were factors contributing to reduced sepsis risk, while gender (=female) was a factor contributing to increased sepsis risk. Under the relative effects, the final predicted value for this patient was 0.002, which is lower than the base value of 0.6211, indicating that this patient belongs to the low-risk category.

**Figures 8B, D** show the waterfall and force plots for another patient with relatively higher risk. Antibiotics (=1), SOFA (=5), nephrotoxic drugs (=1), total serum calcium (=7.5 mg/dL), glucocorticoids (=1), dementia (=1), and RDW (=24.4%) were factors contributing to increased sepsis risk, while pneumonia (=0) and gender (=male) were factors contributing to reduced sepsis risk. Under the relative effects, the final predicted value for this patient was 0.97, indicating a high-risk patient.

### Clinical application

**Figure 9:**
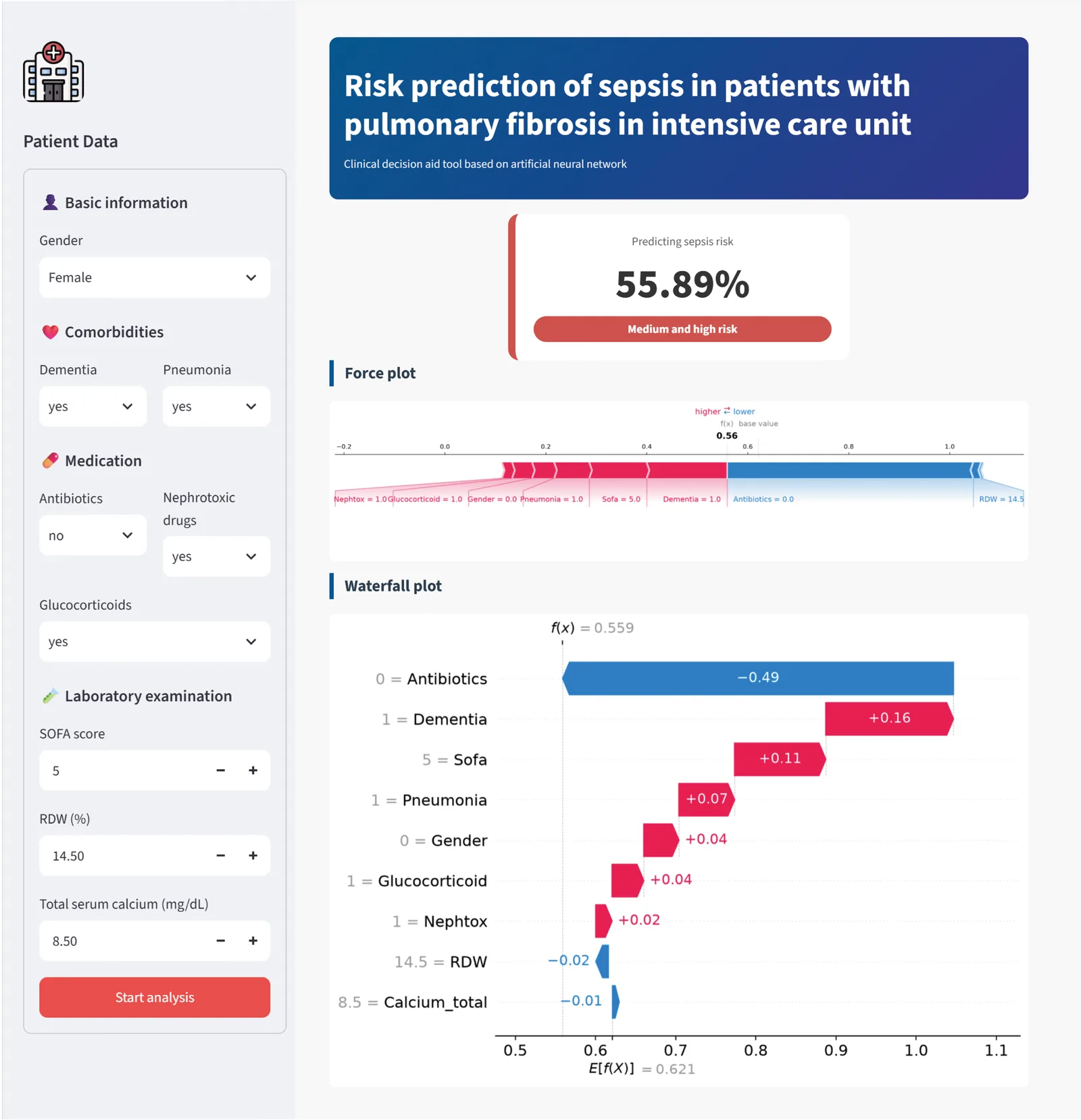
Web interface. The interface is divided into two main sections. Users can enter patient data in the left sidebar. The demographic section includes gender; the laboratory examination section includes RDW and total serum calcium. The clinical status section includes dementia, pneumonia, antibiotics, nephrotoxic drugs, glucocorticoids, and SOFA. After completing all required information, users can click the “Run Analysis” button to view the risk prediction results and the contribution of each feature in the right main panel.

Based on the Streamlit framework in Python, an online prediction platform was built. Users can input demographic data, laboratory test results, treatment measures, and medical history through the interface to achieve rapid risk assessment of sepsis in ICU patients with pulmonary fibrosis, as shown in **Figure 9**. Prediction results are classified into four categories: high risk, medium-high risk, medium risk, and low risk, with corresponding risk probability percentages provided for each category. Meanwhile, based on the SHAP interpretability analysis method, the platform displays the influence of each feature on the prediction outcome, including force plots and waterfall plots. Red indicates that the feature increases the risk of sepsis, while blue indicates that it reduces the risk. For more details on the specific functions and usage of this platform, please visit https://icu-pf-stpqmiinny5hksxxm6cxny.streamlit.app/.

## Discussion

Patients with pulmonary fibrosis in the ICU represent a special population with an extremely severe prognosis. Pulmonary fibrosis is characterized pathologically by progressive interstitial collagen deposition and irreversible gas exchange impairment, which severely limits patients’ compensatory capacity against infectious insults (Warheit-Niemi et al., 2022; Singh et al., 2025). Once sepsis is superimposed, patients with pulmonary fibrosis face a higher risk of mortality and requirement for invasive mechanical ventilation, and acute exacerbation of IPF itself can have systemic consequences, causing extra-pulmonary organ failure (Zhuang et al., 2020). However, previous studies have rarely focused on the ICU population with pulmonary fibrosis, and to date, no study has systematically validated the predictive performance of machine learning models for sepsis occurrence in this specific population across multiple large-scale databases, resulting in a lack of available decision-making tools for early identification and precise intervention in these high-risk patients in clinical practice.

This study is the first to utilize two large intensive care databases, MIMIC-IV and MIMIC-III, enrolling 728 ICU patients with concomitant pulmonary fibrosis, to construct and validate across multiple databases six machine learning models for predicting the probability of sepsis occurrence. The results showed that the ANN algorithm performed optimally, with an AUC of 0.878 in the training set, 0.837 in the internal validation set, and 0.857 in the MIMIC-III external validation set. Given the significant differences between MIMIC-III and MIMIC-IV in data collection time periods, institutional information systems, and population composition, the model maintained an AUC above 0.8 in the external validation set, indicating good generalizability and cross-database robustness. By predicting sepsis risk in ICU patients with pulmonary fibrosis, our model can effectively assist clinicians in making accurate and proactive treatment decisions for such patients at an early stage.

As a feedforward neural network that automatically extracts data features through multiple layers of nonlinear transformation, ANN demonstrates unique advantages in handling complex coupling relationships among high-dimensional clinical variables (Tungushpayev et al., 2025). This supervised learning technique operates at high speed and demonstrates high adaptability and generalization (Ghafariasl et al., 2024). Compared with traditional models that use piecewise linear or discrete rules for fitting, ANN does not require prespecified discrete cut points for variables and can flexibly capture highly coupled nonlinear interaction effects among multiple physiological and laboratory indicators using continuous activation functions (Dreiseitl and Ohno-MaChado, 2002). ANN and optimization frameworks have been applied in a variety of medical clinical tasks (Lai et al., 2023; Ghafariasl et al., 2025). However, the “black-box” nature of ANN models has always been a key obstacle limiting their clinical promotion. To this end, this study introduced SHAP analysis to provide a visual explanation of the model’s prediction logic, presenting the contribution of each feature to the prediction outcome to clinicians in an intuitive manner, thereby bridging the gap between model predictive accuracy and clinical interpretability.

Through Lasso regression, nine consistently crucial clinical features were screened in this study: gender, dementia, pneumonia, antibiotics, nephrotoxic drugs, glucocorticoids, SOFA, RDW, and total serum calcium. SHAP analysis further indicated that SOFA score was the most influential predictor, followed by antibiotics and pneumonia. SOFA, as a widely used tool for assessing organ dysfunction in the ICU, has been established as a core component of the Sepsis-3 definition (Singer et al., 2016). RDW was identified as an important predictor, with substantial literature support in the pulmonary fibrosis population. Nathan et al. analyzed baseline data from 319 IPF patients and showed that the median survival was 43.1 months for those with normal RDW (≤15) compared to only 16.3 months for those with RDW >15 (Gürün Kaya et al., 2021). Moreover, RDW is easily obtainable from routine blood tests, further enhancing the clinical generalizability of the model. The association between total serum calcium and prognosis in pulmonary fibrosis is also supported by evidence. A retrospective cohort study by Lin et al. confirmed that the red blood cell distribution width-to-total calcium ratio had predictive value for in-hospital mortality in ICU patients with interstitial lung disease (Lin et al., 2026).

Pneumonia and antibiotics reflect the dual roles of infection trigger and iatrogenic factors. Pneumonia directly points to the initiating event of sepsis, while antibiotics have a bidirectional effect—they not only reflect pre-existing infectious status but their exposure itself may increase subsequent sepsis risk. Baggs et al. found that patients exposed to high-risk antibiotics during hospitalization had a 65% higher risk of sepsis within 90 days after discharge compared to unexposed patients (Baggs et al., 2018).

Nephrotoxic drugs and glucocorticoids involve medication safety issues in patients with pulmonary fibrosis. Great caution is required when using nephrotoxic drugs in ICU patients with concomitant pulmonary fibrosis, as pulmonary fibrosis itself can adversely affect renal function through the “lung-kidney interaction” (Faubel, 2008). Acute kidney injury can promote lung inflammation and fibrosis through mechanisms such as systemic inflammatory mediator release and Th17 cell infiltration (Mehrotra et al., 2018). The use of nephrotoxic drugs effectively adds a second hit to an already threatened “lung-kidney axis, “ creating a vicious cycle and significantly increasing the risk of sepsis and multiple organ failure (Hoste et al., 2018). A meta-analysis indicated that IPF patients with acute exacerbation who received corticosteroid therapy exhibited higher short-term and long-term mortality rates, suggesting a potential association between glucocorticoids and prognosis in pulmonary fibrosis (Mondoni et al., 2026). Anan et al. also found that early tapering of corticosteroids was associated with favorable outcomes in patients with acute exacerbation of IPF (Anan et al., 2022).

Dementia was identified as a key variable as a comorbidity indicator, reflecting the potential impact of cognitive impairment on disease prognosis. Accumulating evidence suggests that cognitive dysfunction is a common extra-pulmonary manifestation of IPF and may exist even in the early stages of the disease. A prospective observational study by Sharp et al. of non-hypoxemic IPF patients showed that 46.7% of patients had at least mild cognitive dysfunction (Sharp et al., 2016). Studies have shown that cognitive dysfunction is prevalent in IPF patients, with the most common impairments being in working memory, verbal memory, processing speed, and executive function (Mihart et al., 2025). For critically ill patients with comorbid dementia, the prognosis is even more severe. A study by Graversen et al., based on Danish national healthcare data, included 25, 948 hospitalized patients with dementia and pneumonia and found that the 30-day mortality risk was 129% higher than that of patients without dementia (Graversen et al., 2021). Patients with dementia have significantly increased susceptibility to pneumonia and sepsis due to dysphagia, increased aspiration risk, and impaired functional status, while cognitive impairment may affect patients’ cooperation with treatment measures and early symptom recognition.

It is worth noting that female gender was identified as one of the predictive factors contributing to increased sepsis risk in this model. This finding appears inconsistent with previous findings that IPF incidence is higher in males and overall survival is shorter in males (Mulholland et al., 2025). One possible explanation is that this association may partly reflect survival-related bias rather than an intrinsically higher susceptibility to sepsis among female patients. Studies have shown that male IPF patients have more rapid disease progression and poorer prognosis. A multi-database retrospective cohort study by Zaman et al. confirmed that male gender remained an independent risk factor for death after adjusting for age and pulmonary function data (Zaman et al., 2020). This may suggest that male patients with IPF may have a shorter time window from diagnosis to death than female patients, thus facing terminal events earlier. Conversely, female patients may have a relatively longer window for infectious exposure, which could partly explain the higher observed probability of sepsis among female survivors. To further explore this possibility, we conducted an exploratory analysis. The results showed that male patients had a slightly shorter median ICU length of stay than female patients (3.01 vs. 3.11 days, P = 0.894). Similarly, among patients who died during hospitalization, the median time from ICU admission to in-hospital death was slightly shorter in male patients than in female patients (7.32 vs. 7.75 days, P = 0.800). However, these differences were not statistically significant. Therefore, this explanation should be interpreted cautiously as a possible but unproven hypothesis. Further prospective studies, particularly those incorporating time-to-event and competing-risk analyses, are needed to clarify whether sex-related differences in survival duration or ICU exposure time contribute to the association.

The sensitivity analyses further clarified the contribution of variables related to sepsis clinical management to model performance. The finding that model discrimination remained acceptable after the individual exclusion of SOFA score, pneumonia, or antibiotics suggests that the predictive framework was not dependent on any single management-related variable. Meanwhile, the reduced performance observed after simultaneous exclusion of all three variables indicates that these variables provided complementary predictive information and contributed to the enhanced performance of the primary model. Importantly, the fully excluded model still retained moderate discriminatory ability, suggesting that the remaining clinical predictors continued to capture relevant risk information. Therefore, the primary model may be interpreted as a clinically enriched prediction model, whereas the fully excluded sensitivity model provides a more conservative estimate of predictive performance. These findings provide supportive evidence for the robustness of the overall modeling strategy.

Several limitations of this study should be acknowledged. First, the retrospective observational design cannot avoid residual confounding bias and can only establish associations rather than causal relationships between variables. For example, although Lasso screened antibiotics and glucocorticoids as important predictors, this may reflect either the direct biological effects of these drugs or the effect of pre-existing infection or disease severity as confounding factors, and the current analysis cannot fully distinguish between these two interpretations. Second, the model was constructed based on static data from the first 24 hours in the ICU and did not incorporate dynamic changes during treatment, which may contain richer prognostic information. Future studies could explore the incorporation of longitudinal data to capture the dynamic trajectory of sepsis occurrence. Third, although multiple variables from the databases were included in this study, some potentially important predictors, such as specific subtypes of pulmonary fibrosis, percent predicted forced vital capacity, 6-minute walk distance, and other pulmonary function indicators, were severely missing in the databases used, which may limit the comprehensiveness of the model. Finally, because MIMIC-III and MIMIC-IV both originate from BIDMC, future validation using electronic health record data from independent healthcare systems is necessary.

## Conclusion

This study is the first to develop and validate a machine learning model for predicting sepsis in ICU patients with pulmonary fibrosis across multiple databases. The ANN model, combined with SHAP interpretability, provides a reliable decision-making tool for clinical decision support, and its consistency has been verified in two databases, including our internal validation cohort.

## Data Availability

The original contributions presented in the study are included in the article/**Supplementary Material**, further inquiries can be directed to the corresponding author/s.
