# Supplementary material for: Development of interpretable machine learning models for predicting the probability of sepsis in patients with pulmonary fibrosis in the intensive care unit: based on MIMIC-IV and multi-database validation
Source: Front Cell Infect Microbiol. 2026 Sep 7;16:1894489. doi: 10.3389/fcimb.2026.1894489 (PMC13590296; doi:10.3389/fcimb.2026.1894489)
Supplement: Supplementary file 1 [file DataSheet1.pdf]

Supplementary Material

Figure S1.

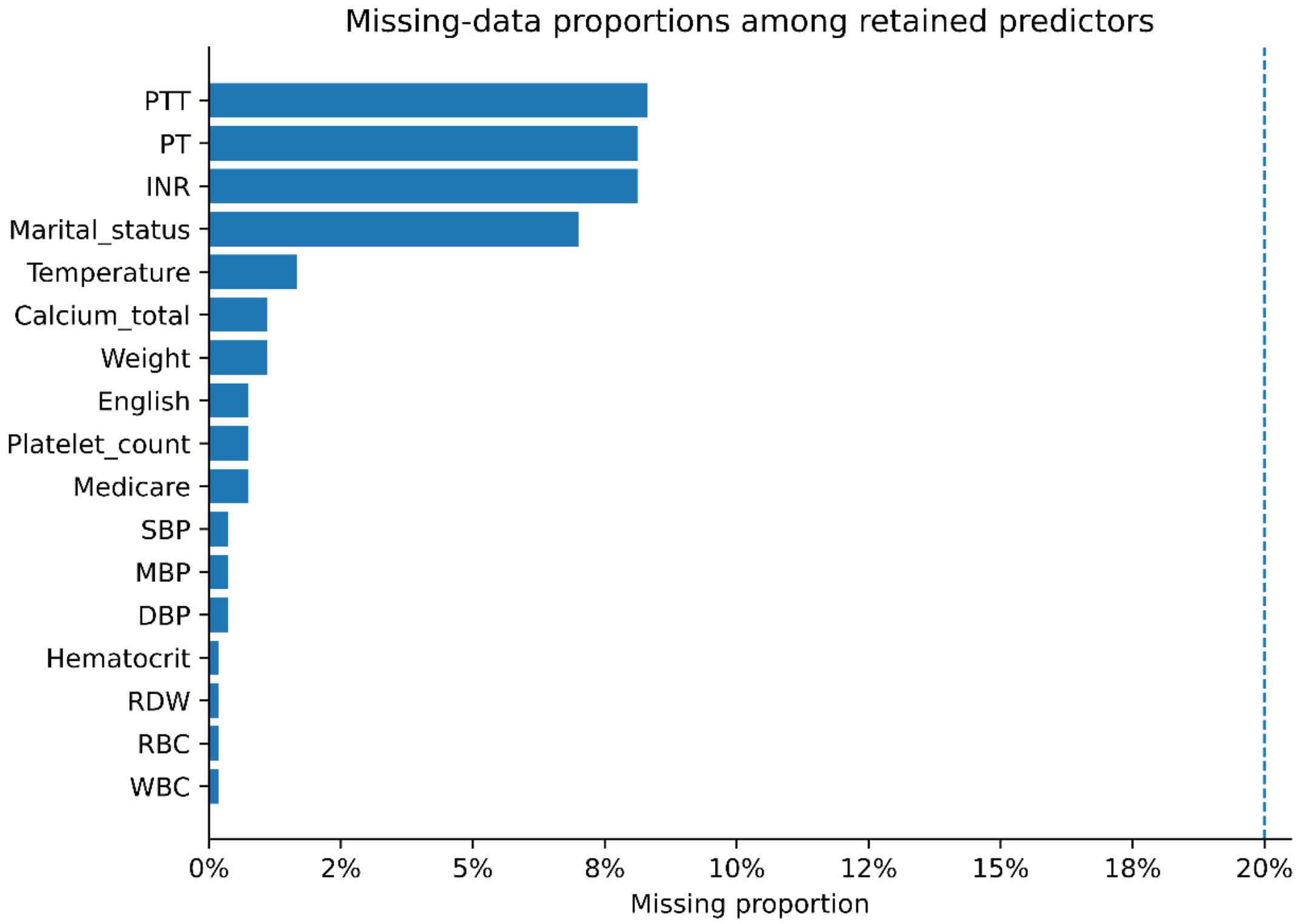

Figure S2.

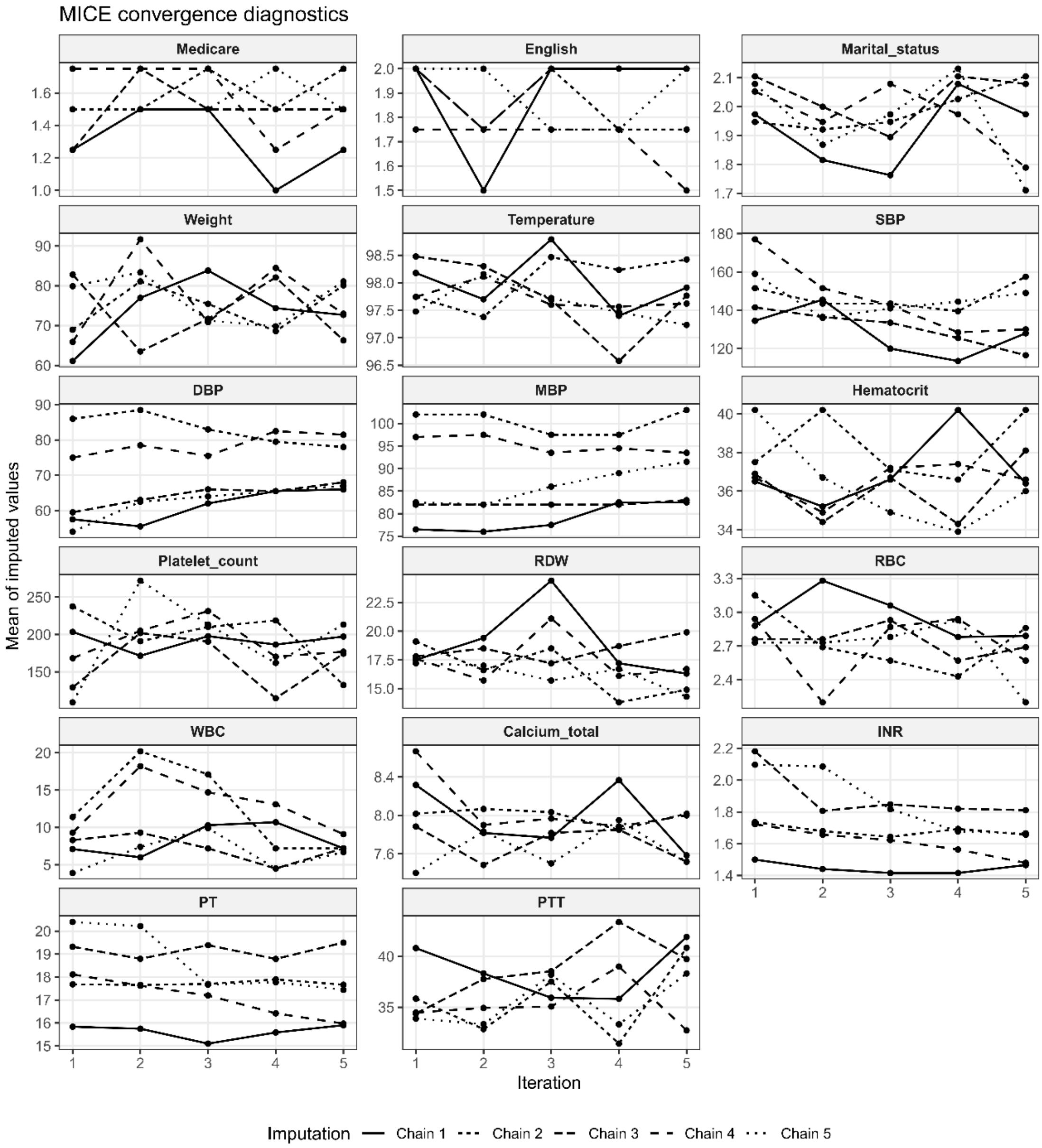
MICE convergence diagnostics
Medicare
English
Marital_status
Weight
Temperature
SBP
DBP
MBP
Hematocrit
Platelet_count
RDW
RBC
WBC
Calcium_total
INR
PT
PTT
Mean of imputed values
Iteration
Imputation
Chain 1
Chain 2
Chain 3
Chain 4
Chain 5

Table S1.

Performance of models in external validation set.

**Models**	**AUC**	**AUC 95% CI Lower**	**AUC 95% CI Upper**	**Accuracy**	**Sensitivity**	**Specificity**	**F1 Score**
LR	0.844	0.778	0.900	0.801	0.880	0.738	0.798
DT	0.870	0.811	0.924	0.903	1.000	0.825	0.902
XGBoost	0.892	0.839	0.938	0.876	0.964	0.806	0.874
LightGBM	0.937	0.900	0.967	0.887	0.940	0.845	0.881
SVM	0.806	0.737	0.866	0.742	0.892	0.621	0.755
ANN	0.857	0.790	0.911	0.812	0.904	0.738	0.811

Figure S3.

Ranking of feature importance by logistic regression.

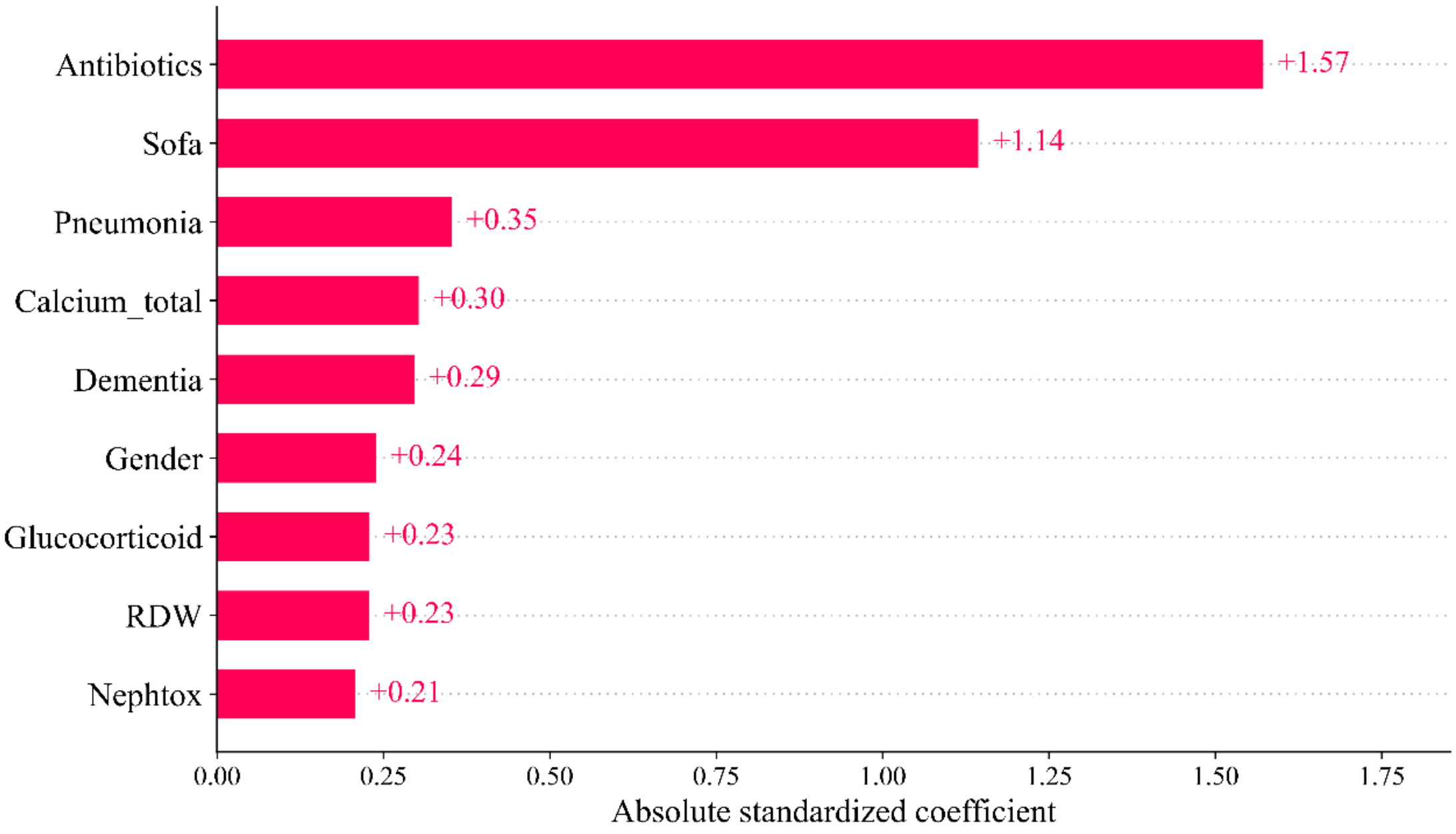

Figure S4.

Ranking of feature importance by Decision Tree.

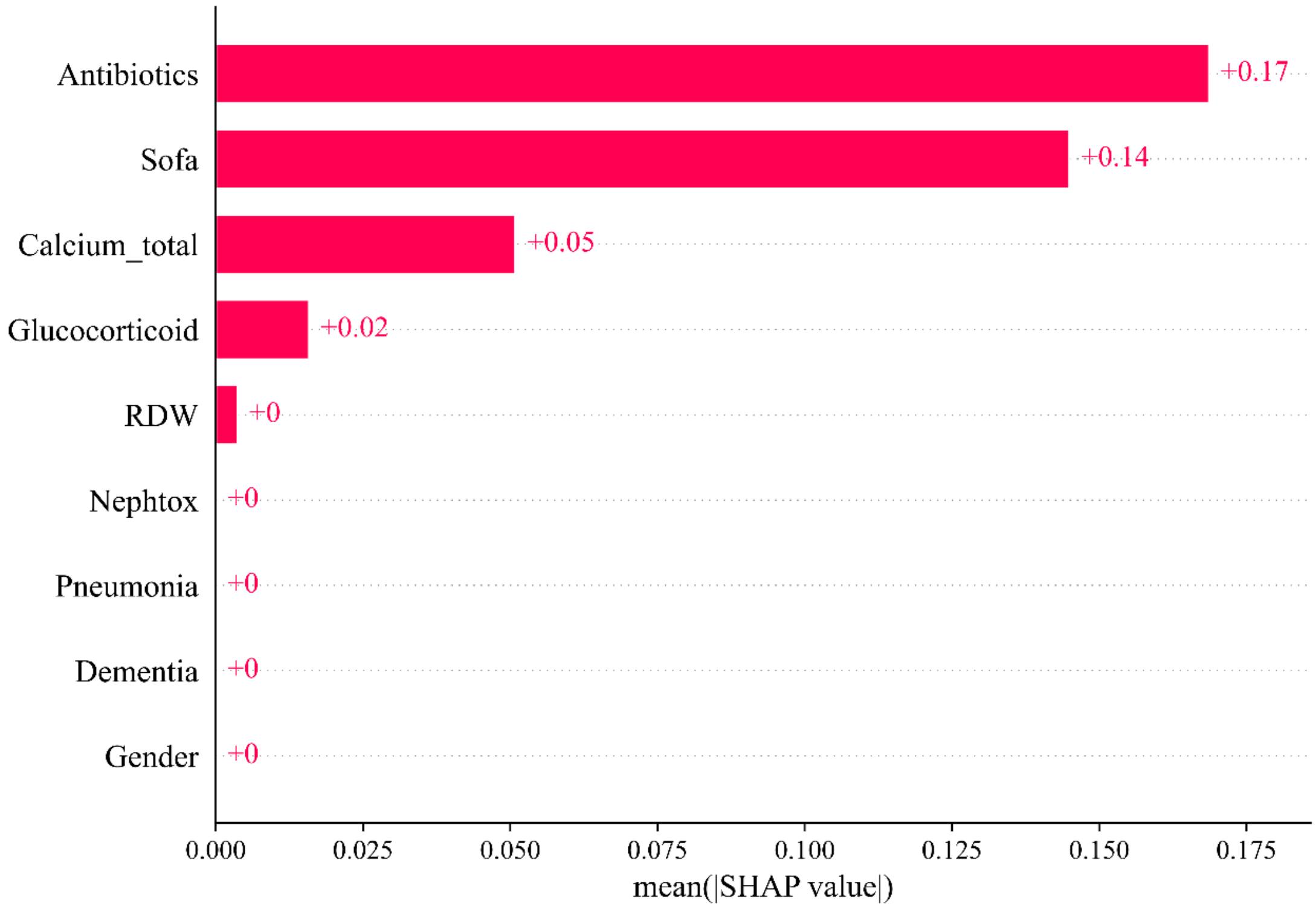

Figure S5.

Ranking of feature importance by XGBoost.

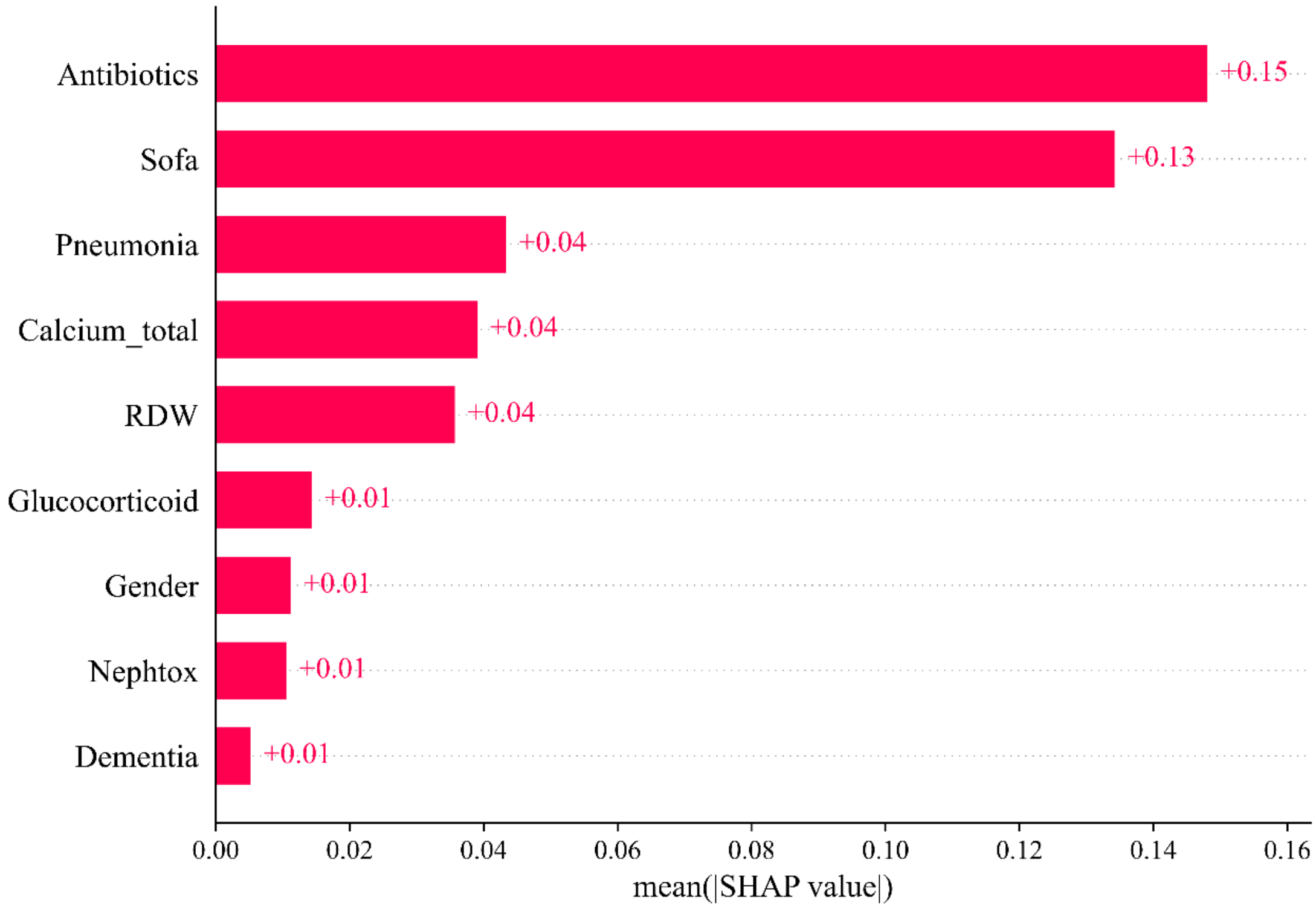

Figure S6.

Ranking of feature importance by LightGBM.

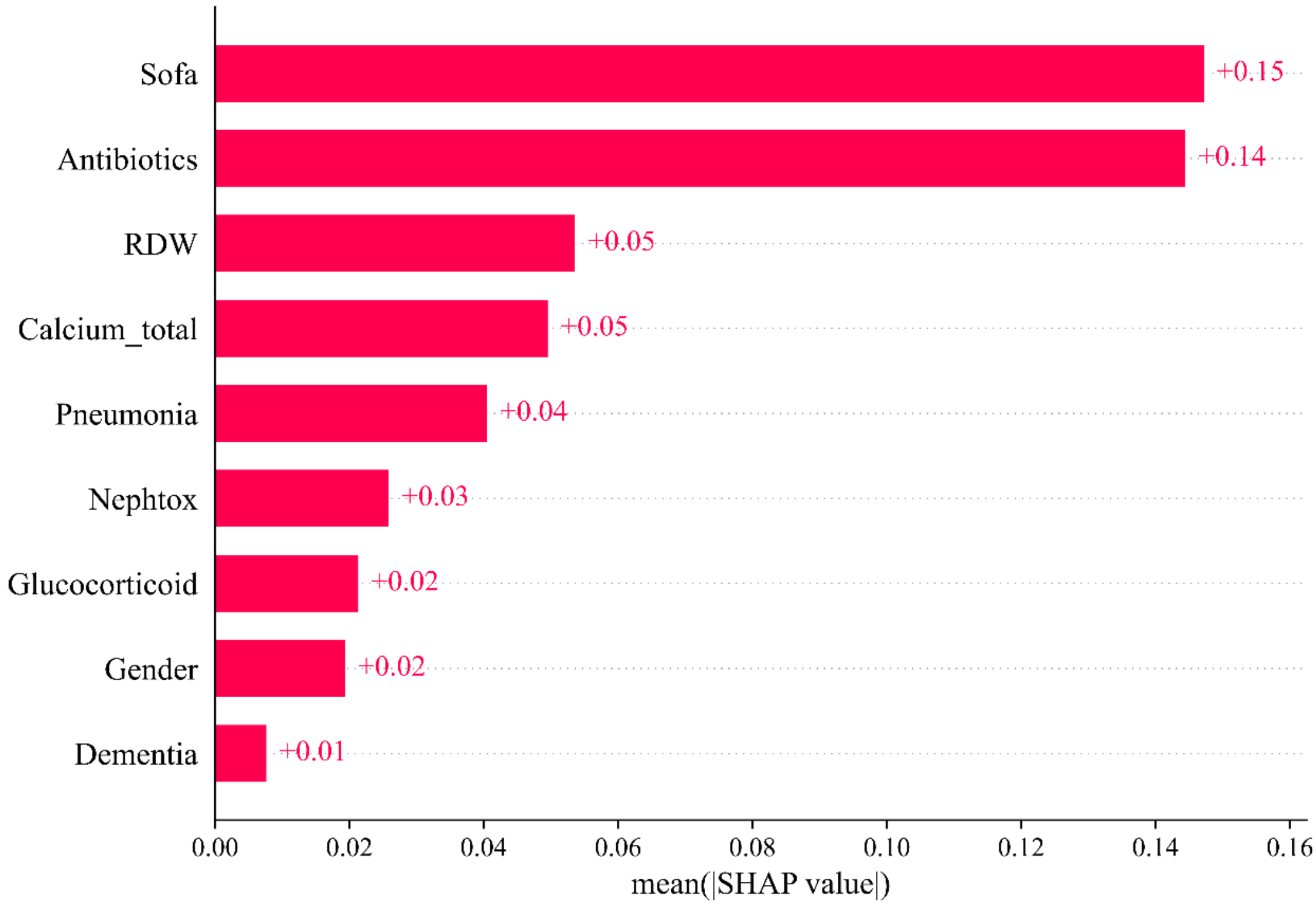

Figure S7.

Ranking of feature importance by SVM.

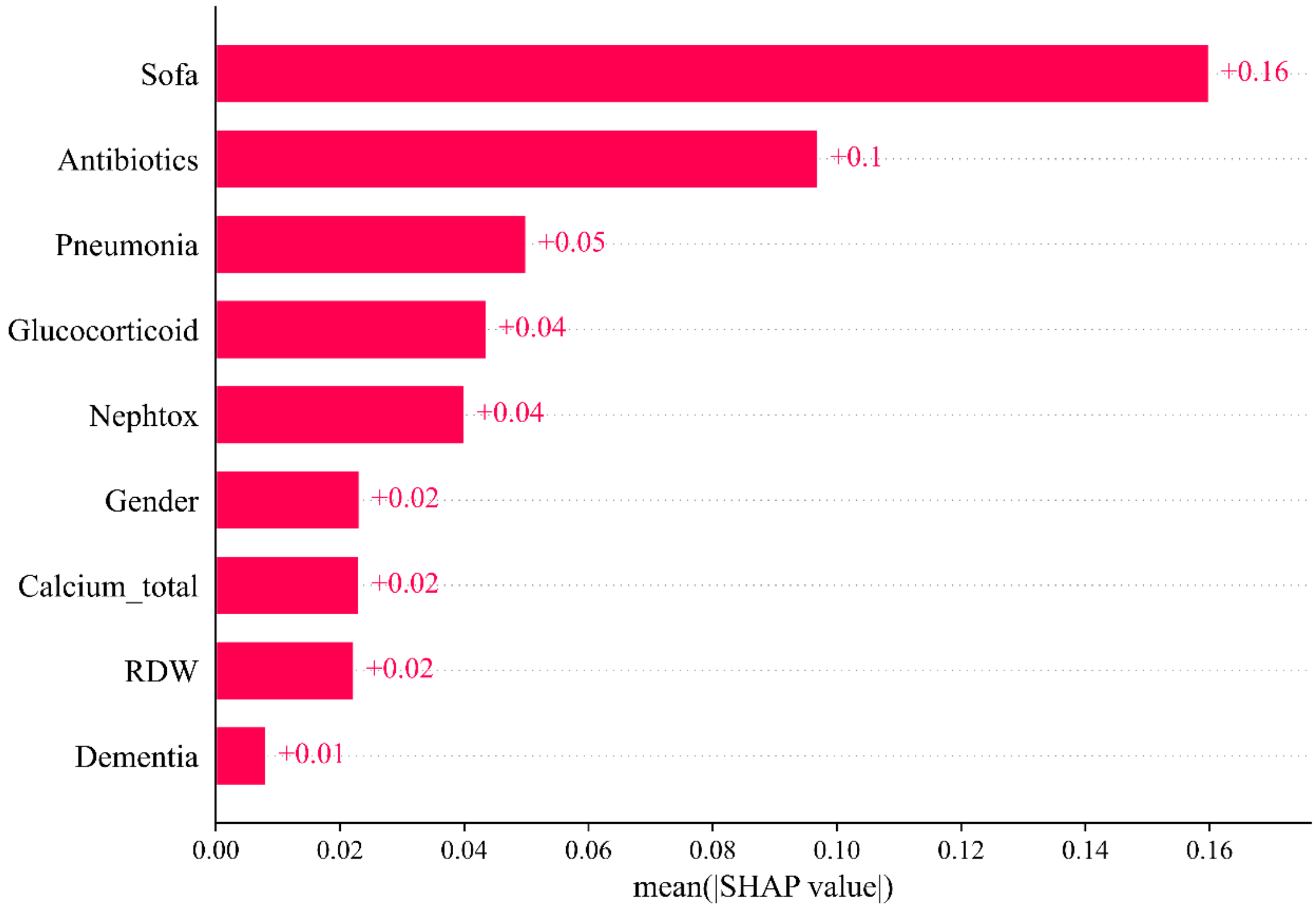

Table S2.

Architecture and training parameters of the ANN model.

Parameter	Value
Algorithm	Artificial neural network / multilayer perceptron classifier
Implementation	MLPClassifier
Hidden architecture	Two hidden layers with 50 neurons each
Activation function	Logistic
Output task	Binary classification
Hyperparameter tuning	GridSearchCV
Cross-validation	10-fold cross-validation
Scoring metric for tuning	AUC
Maximum iterations	500
Decision threshold	0.5
Other parameters	Default settings of MLPClassifier
